# The Future of Bioinspired Innovation: Exploring the Potential of Nanobiomimetics

**DOI:** 10.1021/acs.nanolett.4c02816

**Published:** 2024-09-12

**Authors:** Jan-Henning Dirks, Dorothea Brüggemann

**Affiliations:** † Biological Structures and Biomimetics, Biomimetics-Innovation-Centre, Hochschule Bremen − City University of Applied Sciences, Bremen 28199, Germany; ‡ Biophysics and Applied Biomaterials, Hochschule Bremen − City University of Applied Sciences, 28199 Bremen, Germany

Leonardo da Vinci’s 15th-century design of flying machines, inspired by the anatomy and flight of birds, stands as classic example of organismal biomimetics. His sketches of ornithopters, machines designed to fly by flapping wings like a bird, highlight his deep understanding of aerodynamics and the mechanics of bird flight.^1^ However, the technology of his time, including the lack of lightweight materials and a power source sufficient to achieve lift, rendered the practical realization of his designs unattainable. Da Vinci’s visionary concepts, though not feasible then, inspired future generations to continue exploring the possibilities of human flight, which marked the beginning of modern aerospace technology.

The development of Velcro stands as another quintessential example of bioinspired innovation limited by available technology. Swiss engineer George de Mestral observed in the 1940s how tiny hooks found on the burrs of large burdock adhered to his clothes and his dog’s fur. However, in the mid-20th century it took nearly a decade of research leading to suitable manufacturing techniques before the microstructures required for the Velcro principle could be produced reliably and cost-efficiently on an industrial scale.^2^

These examples illustrate that for many centuries manufacturing processes have limited the application of biomimetic principles in technology. In recent decades, however, remarkable progress has been made in micro- and nanostructuring techniques, combining small scale accuracy with large scale manufacturing. These advances have led to the development of various bioinspired microstructures and self-organizing surface modifications that closely resemble miniature topological features of natural surfaces. The most prominent example is probably the water-repellent lotus leaves, which have been known and used for hundreds of centuries^1^. However, it was not until the 1970s that the principle of hierarchical microstructures for controlling the wettability of functionalized plant surfaces was discovered with improved and easily affordable electron microscopy.^4,5^ Mass-production of such microstructures at economically justifiable costs was even only possible since around the mid-2010s.^6,7^ Similarly, gecko-inspired adhesives mimic the hierarchical structure of gecko feet, utilizing microscale pillars that maximize surface area contact through van der Waals forces, enabling strong, reversible adhesion to various surfaces without leaving residue. As with the lotus effect, for many years the available manufacturing techniques limited the commercial application of this biological principle.^8,9^

More recently (and finally moving toward the nanoscale), the Salvinia effect utilizes a combination of micro- and nanostructures to create superhydrophobic surfaces that mimic the water-repellent properties of the *Salvinia molesta* plant’s leaves. These hair-like structures trap air, significantly reducing the surface’s wetting by water and reducing drag forces.^10,11^ The large-scale production of Salvinia-inspired structures for technical applications is however still a major challenge.^12^ A biological principle functionally limited to the nanoscale are antireflective structures inspired by nanopillars on the eyes of moths.^13^ Although the optical effect is known since the early 1980s, only recently these structures can be mass-produced using commercially available nanoimprinting and semiconductor technology in combination with self-assembly nanolithography.^14,15^

These examples of miniaturization from various technological fields show that successful biomimetic research has long since progressed from organismal principles toward micro- and nanoscale levels and is now at the step of reaching the molecular level, the proximate length scale of most fundamental biological processes (see Figure 1).

**Figure 1:**
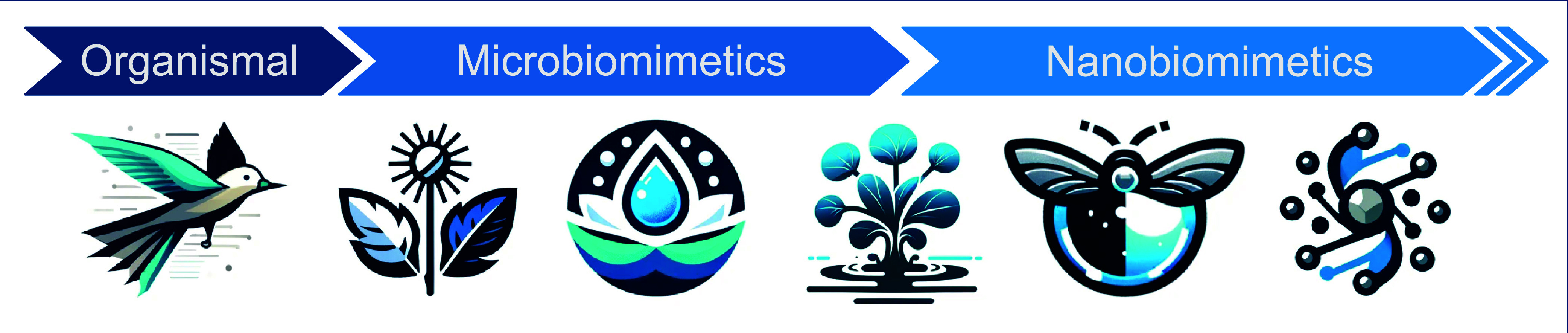
**Selected examples illustrating the miniaturization in bioinspired innovation.** From the organismic level of bird flight, *via* microstructures adapted from lotus and burdock plants, to the combined micro- and nanoscale Salvinia effect to nanoscale moth eye structures and molecular motors.

Within the next years, state-of-the art manufacturing technology will continue to push the boundaries of biomimetics even further, granting researchers the ability to precisely control molecular assemblies with complex hierarchical levels and tailored structure-function relationships and at the same time making them commercially interesting. The ability to transfer complex fundamental molecular biological principles–ranging from protein structures, semipermeable cell membranes to fibrous extracellular matrix–into new technological applications on the molecular length scale will pave the way for a new field of “nanobiomimetics”, possibly the final frontier of this research field.

Nanobiomimetic technologies have the potential to revolutionize various fields. In medicine, nanosized carriers inspired by molecular motors can deliver drugs directly to specific cells, improving efficacy and reducing side effects. Molecular motors are an essential part of every living cell and offer distinct advantages over conventional mechanical motors, particularly in terms of size, efficiency, and adaptability. They convert chemical energy directly into mechanical work, making them extremely energy- and resource-efficient. Molecular motors are also very precise and enable targeted signaling that is crucial for many medical applications like drug delivery or tissue engineering. Additionally, many molecular motors can self-assemble and repair to a certain extent, which increases their longevity and reduces the need for maintenance.^16−18^ Molecular motors are integral to numerous biological functions, including DNA replication, cellular transport and metabolism.^19,20^ However, since their complex fundamental biophysical principles cannot easily be scaled up to the micro- or macrolevel, harvesting their biomimetic potential requires a downscaling of the manufacturing dimensions and thus an increased use of molecular nanotechnology. Such nanobiomimetic molecular machines, that are either built or self-assembled to function at the molecular length scale, can enable countless applications in medicine, biotechnology and smart materials design in the future.^21,22^

On a different length scale, bioinspired nanomaterials can support tissue regeneration by mimicking the extracellular matrix, promoting healing and repair.^23^ Nanoscale sensors embedded in these materials can also detect biomarkers with high sensitivity and thus enable the early diagnosis of various diseases. In biotechnology and materials science, materials inspired by biological self-repair mechanisms can even self-heal and thereby increase product longevity^24^ while adaptive bioinspired materials can change their properties on the nanoscale in response to external stimuli. This special material design is increasingly leading to product innovations such as smart textiles and adaptive coatings.^25^ Furthermore, nanoscale catalysts, i.e. nanozymes, have become attractive alternatives for natural enzymes since they can be easily regulated, have robust activity and show potential for catalytic selectivity when combined with DNA chains, which makes them attractive for applications in environmental monitoring and theranostics.^26,27^ Environmental and energy applications can also benefit from nanobiomimetics, such as filter membranes for water purification that remove impurities at the molecular level to reduce pollution and provide clean water.^28^ Moreover, nanobiomimetic systems can mimic photosynthetic processes to harvest solar energy with high efficiency,^29^ and nanoscale sensors can detect environmental pollutants with high sensitivity, contributing to real-time monitoring of pollution control.^28^ This wide range of applications impressively demonstrates the versatility of industrial processes and materials that will benefit from nanobiomimetics and a trend of miniaturization in the future.

As we continue to push the boundaries of biomimetic research from the micro- to the nanoscale, the potential for groundbreaking technological advancements is immense. The path of miniaturization from Leonardo da Vinci’s ornithopters to modern molecular motors illustrates the transformative power of bioinspired innovation. However, to fully harness the potential of nanobiomimetics, it is crucial to foster interdisciplinary collaboration and education for the next generation of scientists and engineers. Training students in a multidisciplinary environment, where they can learn from biology, chemistry, physics, materials science, and engineering, will equip them with the skills and knowledge necessary to drive innovation in this exciting new research field.

## Footnotes

1 The water-repellent properties of Lotus leaves are already described in the Hindu scripture Bhagavad Gita (BG 5:10): “He that acts in thought of Brahm, Detaching end from act, with act content, The world of sense can no more stain his soul Than waters mar th’ enamelled lotus-leaf.”^3^

## References

[ref1] da Vinci L. Codex on the Flight of Birds - Codice Sul Volo Degli Uccelli, 1505. https://www.loc.gov/item/2021668201/ (accessed 2024-04-26).

[ref2] Strauss S. D. *The Big Idea: How Business Innovators Get Great Ideas to Market*; Kaplan Financial Series; Dearborn Trade Pub., 2002.

[ref3] Arnold E. The Bhagavad-Vita, A Word To The Wise. https://search.ebscohost.com/login.aspx?direct=true&db=nlebk&AN=1622448&site=ehost-live (accessed 2024-04-26).

[ref4] Barthlott W.; Neinhuis C. Purity of the Sacred Lotus, or Escape from Contamination in Biological Surfaces. Planta 1997, 202 (1), 1–8. 10.1007/s004250050096.

[ref5] Barthlott W. Self-Cleaning Surfaces in Plants: The Discovery of the Lotus Effect as a Key Innovation for Biomimetic Technologies. In Handbook of Self-Cleaning Surfaces and Materials; Zhang X., Tryk D., Irie H., Fujishima A., Eds.; Wiley, 2023; pp 359–369. 10.1002/9783527690688.ch15.

[ref6] Zhao X. D.; Xu G. Q.; Liu X. Y. Superhydrophobic Surfaces: Beyond Lotus Effect. In *Bioinspiration*; Liu X. Y., Ed.; Biological and Medical Physics, Biomedical Engineering; Springer New York: New York, NY, 2012; pp 331–378. 10.1007/978-1-4614-5372-7_9.

[ref7] Zhang M.; Feng S.; Wang L.; Zheng Y. Lotus Effect in Wetting and Self-Cleaning. Biotribology 2016, 5, 31–43. 10.1016/j.biotri.2015.08.002.

[ref8] Brodoceanu D.; Bauer C. T.; Kroner E.; Arzt E.; Kraus T. Hierarchical Bioinspired Adhesive Surfaces—a Review. Bioinspir. Biomim. 2016, 11 (5), 051001 10.1088/1748-3190/11/5/051001. 27529743

[ref9] Niewiarowski P. H.; Stark A. Y.; Dhinojwala A. Sticking to the Story: Outstanding Challenges in Gecko-Inspired Adhesives. Journal of Experimental Biology 2016, 219 (7), 912–919. 10.1242/jeb.080085. 27030772

[ref10] Barthlott W.; Mail M.; Neinhuis C. Superhydrophobic Hierarchically Structured Surfaces in Biology: Evolution, Structural Principles and Biomimetic Applications. Philos. Trans. R. Soc. A 2016, 374 (2073), 20160191 10.1098/rsta.2016.0191. PMC4928508 27354736

[ref11] Solga A.; Cerman Z.; Striffler B. F.; Spaeth M.; Barthlott W. The Dream of Staying Clean: Lotus and Biomimetic Surfaces. Bioinspir. Biomim. 2007, 2 (4), S126–S134. 10.1088/1748-3182/2/4/S02. 18037722

[ref12] Kim M.; Yoo S.; Jeong H. E.; Kwak M. K. Fabrication of Salvinia-Inspired Surfaces for Hydrodynamic Drag Reduction by Capillary-Force-Induced Clustering. Nat. Commun. 2022, 13 (1), 5181 10.1038/s41467-022-32919-4. 36056031 PMC9440115

[ref13] Wilson S. J.; Hutley M. C. The Optical Properties of “Moth Eye” Antireflection Surfaces. Optica Acta: International Journal of Optics 1982, 29 (7), 993–1009. 10.1080/713820946.

[ref14] Chen Q.; Hubbard G.; Shields P. A.; Liu C.; Allsopp D. W. E.; Wang W. N.; Abbott S. Broadband Moth-Eye Antireflection Coatings Fabricated by Low-Cost Nanoimprinting. Appl. Phys. Lett. 2009, 94 (26), 263118 10.1063/1.3171930.

[ref15] Diao Z.; Kraus M.; Brunner R.; Dirks J.-H.; Spatz J. P. Nanostructured Stealth Surfaces for Visible and Near-Infrared Light. Nano Lett. 2016, 16 (10), 6610–6616. 10.1021/acs.nanolett.6b03308. 27673379

[ref16] Browne W. R.; Feringa B. L. Making Molecular Machines Work. Nat. Nanotechnol. 2006, 1 (1), 25–35. 10.1038/nnano.2006.45. 18654138

[ref17] Huang T. J.; Juluri B. K. Biological and Biomimetic Molecular Machines. Nanomedicine 2008, 3 (1), 107–124. 10.2217/17435889.3.1.107. 18393670

[ref18] Lancia F.; Ryabchun A.; Katsonis N. Life-like Motion Driven by Artificial Molecular Machines. Nat. Rev. Chem. 2019, 3 (9), 536–551. 10.1038/s41570-019-0122-2.

[ref19] Erbas-Cakmak S.; Leigh D. A.; McTernan C. T.; Nussbaumer A. L. Artificial Molecular Machines. Chem. Rev. 2015, 115 (18), 10081–10206. 10.1021/acs.chemrev.5b00146. 26346838 PMC4585175

[ref20] Zhang L.; Wu H.; Li X.; Chen H.; Astumian R. D.; Stoddart J. F. Artificial Molecular Pumps. Nat. Rev. Methods Primers 2024, 4 (1), 1–21. 10.1038/s43586-024-00291-w.

[ref21] Krause S.; Feringa B. L. Towards Artificial Molecular Factories from Framework-Embedded Molecular Machines. Nat. Rev. Chem. 2020, 4 (10), 550–562. 10.1038/s41570-020-0209-9.

[ref22] Tasbas M. N.; Sahin E.; Erbas-Cakmak S. Bio-Inspired Molecular Machines and Their Biological Applications. Coord. Chem. Rev. 2021, 443, 214039 10.1016/j.ccr.2021.214039.

[ref23] Górnicki T.; Lambrinow J.; Golkar-Narenji A.; Data K.; Domagała D.; Niebora J.; Farzaneh M.; Mozdziak P.; Zabel M.; Antosik P.; Bukowska D.; Ratajczak K.; Podhorska-Okołów M.; Dzięgiel P.; Kempisty B. Biomimetic Scaffolds—A Novel Approach to Three Dimensional Cell Culture Techniques for Potential Implementation in Tissue Engineering. Nanomaterials 2024, 14 (6), 531 10.3390/nano14060531. 38535679 PMC10974775

[ref24] Brooks S.; Roy R.; Dirks J.-H.; Taylor D. A Systematic Study of Biological SE Systems from Complexity and Design Perspectives. Journal of Engineering Design 2023, 34 (11), 897–921. 10.1080/09544828.2023.2266864.

[ref25] Kitchen G.; Sun B.; Kang S. H. Bioinspired Nanocomposites with Self-Adaptive Mechanical Properties. Nano Res. 2023, 17 (2), 633–648. 10.1007/s12274-023-6141-9.

[ref26] Liang H.; Chen X.; Bu Z.; Bai Q.; Liu J.; Tian Q.; Tang Z.; Li S.; Diao Q.; Niu X. When Nanozymes Meet Deoxyribonucleic Acid: Understanding Their Interactions and Biomedical Diagnosis Applications. Interdisciplinary Medicine 2024, 2 (2), e20230057 10.1002/INMD.20230057.

[ref27] Margret A A.; R P. Enzyme-like Activity of Nanozymes, the Enzyme Mimics. In Nano-Enzyme Incorporated Particles; Elsevier, 2024; Chapter 3, pp 87–112. 10.1016/B978-0-443-18810-7.00003-X.

[ref28] Ling S.; Qin Z.; Huang W.; Cao S.; Kaplan D. L.; Buehler M. J. Design and Function of Biomimetic Multilayer Water Purification Membranes. Sci. Adv. 2017, 3 (4), e1601939 10.1126/sciadv.1601939. 28435877 PMC5381955

[ref29] Kathpalia R.; Verma A. K. Bio-Inspired Nanoparticles for Artificial Photosynthesis. Materials Today: Proceedings 2021, 45, 3825–3832. 10.1016/j.matpr.2021.03.214.

